# Trait-based model development to support breeding programs. A case study for salt tolerance and rice

**DOI:** 10.1038/s41598-017-04022-y

**Published:** 2017-06-28

**Authors:** Livia Paleari, Ermes Movedi, Roberto Confalonieri

**Affiliations:** 1 University of Milan, DISAA, Cassandra lab, via Celoria 2, 20133 Milano, Italy; 2 University of Milan, DEMM, Cassandra lab, via Celoria 2, 20133 Milano, Italy

## Abstract

Eco-physiological models are increasingly used to analyze G × E × M interactions to support breeding programs via the design of ideotypes for specific contexts. However, available crop models are only partly suitable for this purpose, since they often lack clear relationships between parameters and traits breeders are working on. Taking salt stress tolerance and rice as a case study, we propose a paradigm shift towards the building of ideotyping-specific models explicitly around traits involved in breeding programs. Salt tolerance is a complex trait relying on different physiological processes that can be alternatively selected to improve the overall crop tolerance. We developed a new model explicitly accounting for these traits and we evaluated its performance using data from growth chamber experiments (e.g., R^2^ ranged from 0.74 to 0.94 for the biomass of different plant organs). Using the model, we were able to show how an increase in the overall tolerance can derive from completely different physiological mechanisms according to soil/water salinity dynamics. The study demonstrated that a trait-based approach can increase the usefulness of mathematical models for supporting breeding programs.

## Introduction

One of the key steps in the planning of breeding programs is the definition of ideotypes able to assure high and stable yields in target areas^1^. An ideotype is a combination of traits that makes a crop suited to the edaphic, climatic and management factors defining a specific agronomic context. However, exploring *in vivo* the deep interaction between those factors and plant genotypes is expensive and time-consuming, being genotype responses nonlinear and the heterogeneity in the environmental and management factors huge. Moreover, one of the priorities for the analysis is trying to account for future climate conditions, but understanding how genotypes would behave in a changing climate remains a challenge^2^.

Given their capability of interpreting genotype (G) × environment (E) × management (M) interactions, crop models are increasingly considered as powerful tools to support breeding activities^3, 4^. Representing genotype features via model parameters, indeed, crop models can be used to answer the “what if” question when the potential impact of varying one or more plant traits is under evaluation^5^. This kind of analysis can involve current conditions and climate change scenarios as well as entire production districts, thus allowing to effectively exploring both spatial and temporal heterogeneity^6^. Moreover, physiologically sound crop models have the potential to integrate the effect of genes or QTLs across different hierarchical levels of organization of biological systems, thus providing insight into their impact at crop scale^3, 7^.

Despite this potential, model development in last decades has been mainly driven by the need of defining management strategies and agricultural policies, and this limited – although to a different extent – their suitability for ideotyping studies^8, 9^. Model parameters do not always have a biological meaning and, even when they have, relationships between model parameters and plant traits are often unclear. This could make the model-based definition of putative ideotypes a speculative exercise^10^.

Dissection and modelling of physiological processes explicitly targeting specific traits of interest within ongoing breeding programs could represent a solution to overcome these limitations. In this way, the overall performance of modelled genotypes would be a consequence of dynamics modulated by variations in the values of parameters that would directly represent plant traits breeders are working on. The resulting *in silico* ideotypes would thus provide clear indications about putative traits for crop improvement, especially when context-specific dependencies play a key role in determining the optimal value of traits contributing to complex plant responses, like in the case of tolerance to abiotic stressors^11^.

With more than 830 million hectares of salt-affected soils, salinity is one of the major environmental stress limiting agricultural production worldwide^12^. Moreover, soil salinization is further increasing^13^ because of both human activities (e.g., inappropriate irrigation practices) and natural causes (e.g., tsunamis), with the latter being exacerbated by climate change^14^. Despite rice is one of the most sensitive crop to salt stress^15, 16^, its frequent cultivation in coastal areas and river deltas increases it exposure because of recurrent flooding and seawater intrusion. For these reasons, ongoing rice breeding activities target different tolerance traits: (i) reduction of shoot sodium (Na^+^) uptake^17, 18^; (ii) sequestration of Na^+^ into structural tissue^19, 20^; (iii) compartmentation of Na^+^ into senescent leaves^21^, (iv) higher leaf tissue tolerance via sequestration of toxic ions into the vacuole and synthesis of osmoprotectants^22^, (v) and higher tolerance to salt-induced sterility^23^. These traits rely on different genetic basis^15^ and can be combined in the same genotype or singularly introduced in commercial varieties even via non-GM technologies (i.e., using marker-assisted selection)^24^. Since the effectiveness of these traits varies according to the environmental context, breeding programs should target these “component traits” and not the overall performance at crop level^25, 26^.

Although models reproducing crop response to salt stress are available^27, 28^, they mainly focus on the effect of salinity on soil osmotic potential, without explicitly considering the toxic effect of Na^+^ in plant tissues which, instead, is a key component of salt stress^15, 17^. In these approaches, plant tolerance is accounted for via few empirical parameters directly linking plant response (in terms of yield or overall growth rate) to soil salinity, without taking into account the physiological traits at the basis of such response. Therefore, these models cannot be considered as suitable to design ideotypes actually relying on the real tolerance traits identified by breeders.

The objectives of this study were (i) building a new model for the toxic effect of Na^+^ on rice by explicitly taking into account the tolerance traits breeders are working on, and (ii) presenting a case study on ideotype design targeting production districts in California and Greece.

## Results and Discussion

### A new model for salt stress built around actual plant traits

**Figure 1:**
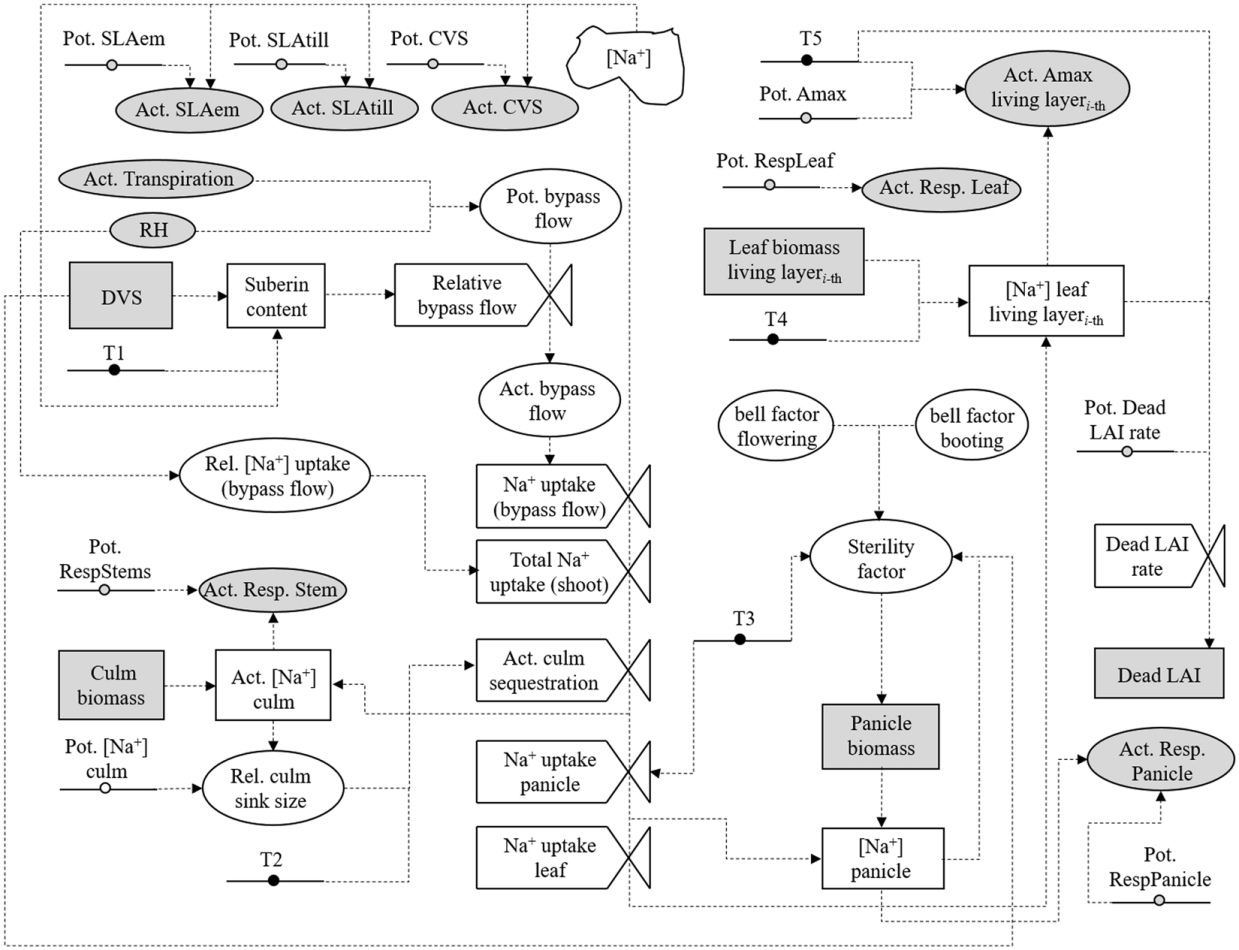
Flowchart of the new model for the impact of salt stress on rice growth. T1, T2, T3, T4, and T5 refer, respectively, to the traits involved with the capability of limiting shoot Na^+^ uptake, sequestrating Na^+^ into the culm base, reducing salt-induced spikelet sterility, storing toxic ions in senescent leaves, and decreasing the toxicity of Na^+^ in photosynthetically active leaves. Grey items represent coupling points between the salinity model and the crop simulator. White items represent variables and parameters of the salinity model. Black items represent parameters corresponding to traits directly involved with tolerance to salinity. Symbols meaning:  represents the main driving variable for salinity-related processes;  identifies parameters;  and  refer to state and rate variables, respectively;  indicates a process.

A new model for the salt stress on rice was developed by directly targeting tolerance traits breeders are working on (Fig. 1). In the following equations, terms with the prefix (T-) refer to traits (Table 1).

**Table 1:** Plant traits, corresponding model parameters and distribution means. Distributions were assumed as normal and standard deviations were set to 5% of the mean values^59^.

Trait	Parameter	Acronym	Units	Mean
Reduction of shoot sodium uptake	Bypass flow when the suberin content is maximum	(T1)RRBF_min_	%	5
	Suberin Deposition Efficiency	(T1)SubDepEff	—	0.62
	Maximum Suberin Content	(T1)SCmax	mg g^−1^	30
Sequestration of sodium in structural tissues	Potential culm sequestration rate	(T2)PotCSeq	mg plant^−1^	0.029
	Maximum culm sodium concentration	(T2)[Na^+^]culmMax	mg g^−1^	26
Tolerance to salt-induced sterility	Susceptibility to salt-induced sterility	(T3)SuscSt	—	0.00135
	Sodium translocation factor to panicle	(T3)Na^+^ToPan	—	0.2
Compartmentation of sodium into senescent leaves	Sodium partitioning capability to older leaves	(T4)PartCap	—	0.7
Tissue tolerance	Threshold leaf sodium concentration	(T5)ThreshL	mg g^−1^	1.5
	Critical leaf sodium concentration	(T5)CritL	mg g^−1^	35

#### Shoot Na^+^ uptake

The plant capability to reduce the shoot Na^+^ uptake relies on a “root filter”^29^ that prevents Na^+^ from entering the roots and getting translocated, via the xylematic stream, to the shoot^26^. The root filter can be more or less pronounced, leading to the identification of “excluder” genotypes as opposite to varieties less effective in preventing Na^+^ from entering the xylem stream^17^.

From a physiological standpoint, the root filter is made of two components: morphological barriers reducing the apoplastic entry of Na^+^ (bypass flow), and channels in the plasma membrane of root epidermal/cortical cells that mediate the selective and non-selective transport of Na^+ ^^18, 30^. Although by-pass flow accounts for 22–35% of the total Na^+^ uptake in rice^17, 31^, it is a key responsible for the differences in the degree of salt tolerance in rice genotypes. This makes the reduction of bypass flow a promising trait for increasing salt tolerance in rice^17, 32^. For this reason and despite its relative importance compared to the other pathway, we decided to focus on by-pass flow to model genotypic differences, given its prominent role in rice breeding activities^17^. The amount of water potentially flowing through the apoplastic pathway (*Jv*_*B*_, mm day^−1^) is alculated according to the following equation:

1 $$J{v}_{B}=\frac{T{r}_{act}\cdot RJ{v}_{B}}{100}$$

where *Tr*_*act*_ (mm day^−1^) is the actual transpiration and *RJv*_*B*_ (%) is the percentage of water uptake through the apoplastic pathway, estimated as a function of air relative humidity (RH; %) according to Faiyue *et al*.^31^ and Steudle *et al*.^33^:

2 $$RJ{v}_{B}=-0.0275\cdot RH+3.92$$

The development of Casparian bands and the deposition of suberin lamellae in the root exo- and endodermis reduce the water transport through the apoplast and thus the bypass flow-Na^+^ uptake^34^. The relative bypass flow (*RRBF*, unitless, 0–1) is thus calculated as a function of the root suberin content^35^:

3 $$RRBF=\frac{1}{100}\cdot (T1)RRB{F}_{min}^{\frac{SC-S{C}_{min}}{(T1)SCmax-S{C}_{min}}}$$

where (*T*1)*RRBF*_*min*_ (%) is the bypass flow when the suberin content is maximum; *SC* (mg g^−1^) is the root suberin content*;* (*T*1)*SCmax* (mg g^−1^) is the maximum suberin content; *SC*_*min*_ (mg g^−1^) is the minimum root suberin concentration at which bypass flow starts to be reduced. The root suberin content is derived as a function of plant age (equation 4) and of the genotype sensitivity to salinity (equation 5)^34, 36^:

4 $$SC=(T1)SCmax\frac{DVS\cdot (0.5\cdot (1-{F}_{sc})+1)}{(1-{F}_{sc})+DVS}$$

5 $${F}_{sc}=\frac{{[N{a}^{+}]}_{ext}}{Max\,{[N{a}^{+}]}_{ext}}\cdot \frac{1}{(1-(T1)SubDepEff)}$$

where *DVS* (unitless; 0–2) is a SUCROS-type development stage code (0: emergence; 1: anthesis; 2: maturity); *F*_*sc*_ (unitless; 0–1) is deposition of suberin in response to salinity; (*T*1)*SubDepEff* (unitless; 0–1) is the suberin deposition efficiency (genotype specific)*;* [*Na* ^*+*^]_*ext*_ and *Max*[*Na* ^*+*^]_*ext*_ (mM) are, respectively, the actual and maximum (at which the suberin deposition is maximum) Na^+^ concentrations in the external medium. Therefore, the amount of Na^+^ actually delivered to the shoot via bypass-flow (*NaUptake*_*AP*_, mg ha^−1^) is:

6 $$NaUptak{e}_{AP}=J{v}_{b}\cdot RRBF\cdot \,{[N{a}^{+}]}_{ext}$$

The fraction of bypass Na^+^ on the total Na^+^ uptake (*RNaUptake*_*AP*_; %) is derived analogously to equation (2)^31^. Finally, the total amount of Na^+^ daily entering the shoot (*NaUptake*, mg ha^−1^) is derived according to equation (7):

7 $$NaUptake=NaUptak{e}_{AP}\frac{100}{RNaUptak{e}_{AP}}$$

#### Sequestration of Na^+^ into structural/senescent organs

To reduce the amount of Na^+^ reaching the leaves, plants have developed mechanisms to accumulate toxic ions in the tissues of culm base and leaf sheath^19, 20^. The former is estimated considering genotypic differences and feedback mechanisms triggered by the amount of Na^+^ already stored in culms^29^. The actual culm sequestration rate (*ActCulmSeqRate*; mg ha^−1^) is derived as:

8 $$ActCulmSeqRate=(T2)PotCSeq\cdot D\cdot \{1-[0.08\cdot ({(RelSinkSize+0.1)}^{-1.13}-0.08)]\}$$

where (*T*2)*PotCSeq* (mg plant^−1^) is the potential capability of the genotype to sequestrate Na^+^ in culms; D is the plant density (plant ha^−1^); *RelSinkSize* (unitless, 0–1) is a dynamic sink capacity of culm for Na^+^ sequestration accounting for feedback mechanisms (equation 9), with (*T*2)[*Na* ^*+*^]*culmMax* and [*Na* ^*+*^]*culmAct* (mg g^−1^) being the maximum and actual Na^+^ concentration in culms, respectively.

9 $$RelSinkSize=1-\frac{{[N{a}^{+}]}_{culmAct}}{(T2)[N{a}^{+}]culmMax}$$

The amount of Na^+^ daily reaching the panicle (*NaPanicle*, mg ha^−1^) is derived as a function of the Na^+^ not sequestrated in culms and of the transport of photosynthates to panicles:

10 $$NaPanicle=\{\begin{array}{cc}ParP\cdot (T3)N{a}^{+}ToPan\cdot (NaUptake-ActCulmSeqRate) & 0.6\le DVS\le 2\\ 0\quad \quad \quad \quad \quad \quad \quad  & elsewhere\end{array}$$

where *ParP* (unitless) is the relative amount of photosynthates daily partitioned to panicles and (*T*3)*Na* ^*+*^ *ToPan* (unitless; 0–1) is the factor for Na^+^ translocation to panicles.

The amount of Na^+^ daily reaching leaves (*Na*_*leaves*_, mg ha^−1^) is the difference between total Na^+^ uptake in shoots and the amounts of Na^+^ sequestrated in the culms and partitioned to panicles. Plants tend to accumulate Na^+^ in the oldest leaves to preserve photosynthetically active tissues from toxic ions^19, 30^. This is represented using equation (11):

11 $$NaUptake{L}_{i}=\frac{N{a}_{leaves}}{{\sum }_{i=1}^{N}[{(1-(T4)PartCap)}^{[\frac{(i-1)}{(N-1)}]}]}\cdot {(1-(T4)PartCap)}^{[\frac{(i-1)}{(N-1)}]}$$

where *NaUptakeL*_*i*_ (mg ha^−1^) is the Na^+^ delivered to the *i*th canopy layer (starting to count from the bottom); (*T*4)*PartCap* (unitless; 0–1) is the genotype capability of partitioning Na^+^ to oldest leaves; *N* is the number of living canopy layers. To account for this heterogeneity in Na^+^ accumulation in leaves of different ages, the model for salt stress should be coupled with a crop model providing a multilayer canopy structure.

#### Impact of Na^+^ on photosynthesis, leaf senescence and spikelet sterility

The Na^+^ effect on photosynthesis^37^ and leaf senescence^38, 39^ depends on the genotype ability to sequestrate toxic ions in the vacuole and to synthesize osmolytes to counterbalance the osmotic pressure. The stress factor for photosynthesis (*RPn*, unitless 0–1) – also used to increase senescence – is derived using equation (12):

12 $$RPn={[(\frac{[N{a}^{+}]{L}_{i}-{[N{a}^{+}]}_{leafmin}}{(T5)ThreshL-{[N{a}^{+}]}_{leafmin}})\cdot (\frac{(T5)CritL-[N{a}^{+}]{L}_{i}}{(T5)CritL-(T5)ThreshL})]}^{{(\frac{(T5)CritL-(T5)ThreshL}{(T5)ThreshL-{[N{a}^{+}]}_{leafmin}})}^{C}}$$

where [*Na* ^+^]*L*_*i*_ (mg g^−1^) is the Na^+^ concentration in leaves at the *i*th canopy layer; [*Na* ^*+*^]_*leaf min*_ (mg g^−1^) is the Na^+^ concentration in unstressed leaves; (*T5*)*ThreshL* (mg g^−1^) is the Na^+^ concentration above which salt stress starts; (*T*5)*CritL* (mg g^−1^) is the Na^+^ concentration at which photosynthesis becomes null; *C* is a shaping coefficient.

Na^+^ also increases maintenance respiration due to the high metabolic costs of the processes of ion exclusion, vacuolar compartmentation and synthesis of osmolytes^40^. The factor increasing maintenance respiration in leaves (*MRespF*, unitless, 0–1) is thus:

13 $$MRespF=\frac{{[N{a}^{+}]}_{leaves}-{[N{a}^{+}]}_{Thresh}}{{[N{a}^{+}]}_{Crit}-{[N{a}^{+}]}_{Thresh}}$$

where [*Na* ^*+*^]_*Crit*_ (set to 3 mg g^−1^) is the Na^+^ concentration at which respiration is double; [*Na* ^*+*^]_*Thresh*_ (set to 0.5 mg g^−1^) is the Na^+^ concentration at which maintenance respiration starts to be affected; [*Na* ^*+*^]_*leaves*_ (mg g^−1^) is the average concentration in leaves (weighted for layers’ biomass). The same function is used for the increased maintenance respiration in culms.

Salt stress also affects spikelet sterility^23^ according to panicle Na^+^ concentration and plant susceptibility, the latter depending on the genotype and phenological stage (equation 14).

14 $$SterilityF=\{\begin{array}{cc}(T3)SuscSt\cdot {[N{a}^{+}]}_{panicle}\cdot bellF\, & 0.6\le DVS\le 1.1\\ 0\quad \quad \quad \quad \quad \, & elsewhere\end{array}$$

where *SterilityF* (unitless, 0–1) is the factor reducing spikelet fertility due to salt stress; (*T*3)*SuscSt* (unitless; 0–1) represents the genotype susceptibility; *bellF* (unitless; 0–1) is a factor modulating susceptibility according to the within- and between-plant heterogeneity phenological development^41^. The *bellF* is calculated considering two phenological stages of maximum susceptibility to abiotic stress-induced sterility: booting (microsporogenesis; DVS = 0.8) and flowering (DVS = 1). *SterilityF* is then used to reduce the amount of photosynthates daily partitioned to panicles.

The reduction of growth due to the osmotic potential in the external medium^15^ is derived by reducing leaf area expansion^42^ (equation 15) and culm growth^38^ (equation 16) because of limitations to cell wall extension caused by a reduced cell water uptake^43^.

15 $$SLAstress=-0.0002\cdot {[N{a}^{+}]}_{ext}^{2}+0.008\cdot {[N{a}^{+}]}_{ext}+1$$

16 $$CVSstress=0.0002\cdot {[N{a}^{+}]}_{ext}^{2}-0.024\cdot {[N{a}^{+}]}_{ext}+1$$

where *SLAstress* (unitless, 0–1) is the reduction factor for specific leaf area (SLA; m^2^ kg^−1^); *CVSstress* (unitless, 0–1) is the reduction factor for culm growth; ${[N{a}^{+}]}_{ext}$.(mM) is the Na^+^ concentration in the external medium.

The salinity model was coupled to the WOFOST model as modified by Stella *et al*.^44^, to benefit from an explicit multi-layer canopy representation. This version of WOFOST, like the other versions available, does not include algorithms for salt stress. The resulting modelling solution was evaluated using the growth chamber datasets described below and then used for the ideotyping study.

### Model evaluation

**Figure 2:**
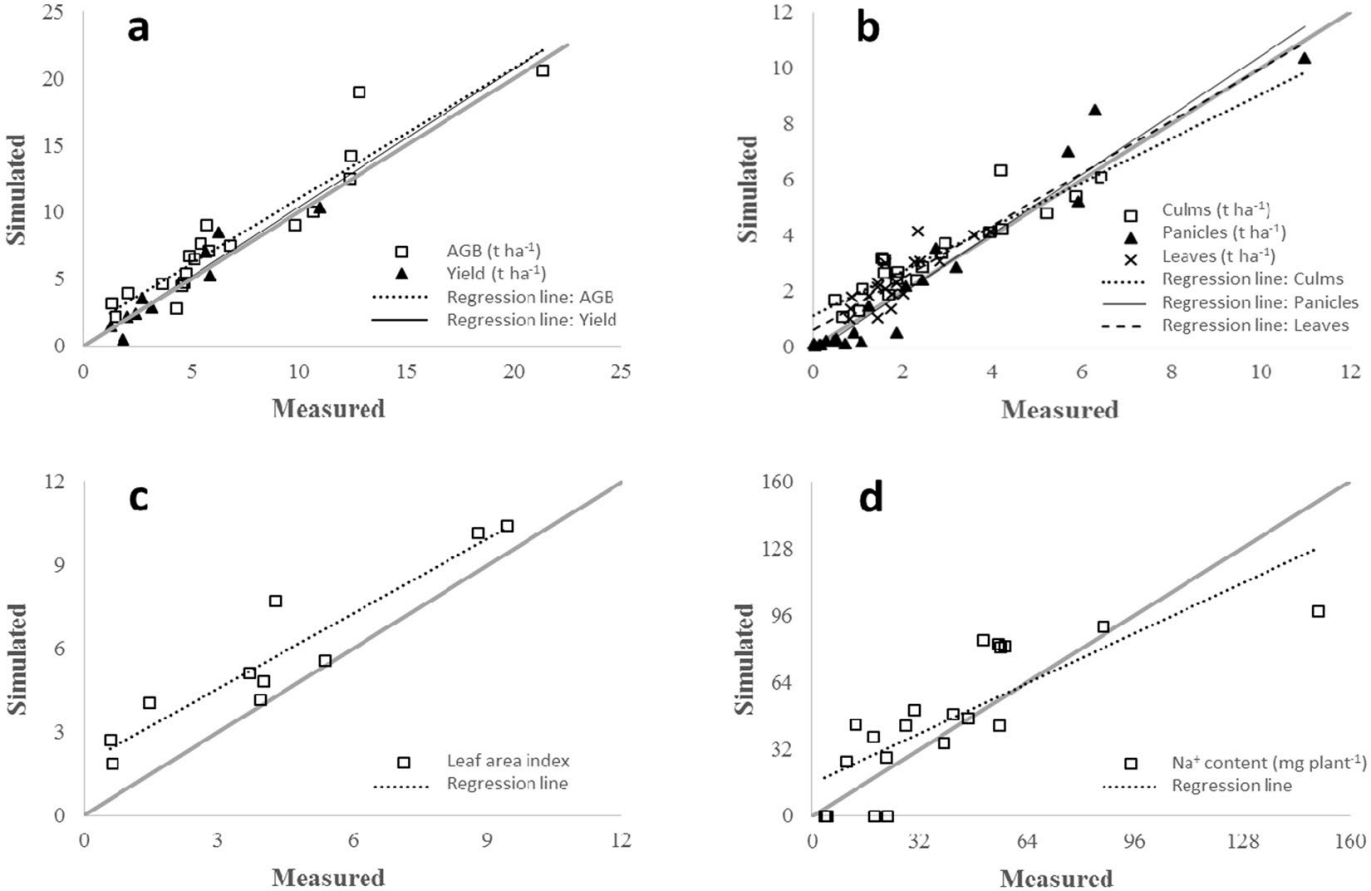
Measured and simulated values of (**a**) aboveground biomass (AGB) and yield, (**b**) biomass of culms, leaves and panicles, (**c**) leaf area index, and (**d**) plant sodium content. The grey line indicates the 1:1 agreement between measured and simulated data.

The agreement between observed and simulated values of aboveground biomass, yield, biomass of culms, leaves and panicles, leaf area index and plant sodium content is shown in Fig. 2. In general, the model showed good performances in reproducing the impact of salt stress on aboveground biomass accumulation and yield (Fig. 2a), with relative root mean square error (RRMSE; %; 0 to +∞, optimum 0) equal to 28.0% and 23.8%, respectively. Good values for these two variables were achieved also for R^2^ (0.89 and 0.90) and modelling efficiency (EF; −∞ to +1, optimum +1)^45^: 0.83 and 0.87. The values of coefficient of residual mass (CRM; −∞ to +∞; optimum = 0)^46^ close to zero highlighted the absence of systematic over- or underestimations.

Good performances were achieved also for the simulation of the biomass of culms, leaves, and panicles (Fig. 2b), with RRMSE never exceeding 36% and values of R^2^ ranging between 0.74 (leaves) and 0.94 (panicles). Regardless of the organ, EF was always positive. Concerning the simulation of panicles weight, the model showed a slight tendency to underestimate the values at heading, likely because of the observed heterogeneity in tiller development. An opposite behavior (slight overestimation around heading) was instead observed for culm and leaf biomass. In this case, the reason is likely an underestimation of the impact of the osmotic component of salt stress on leaf area expansion and tiller development, which are only implicitly reproduced in the current version of the modelling solution, since the crop model used does not explicitly simulate tillering and leaf size. Future model improvements could thus refer to the implementation of dedicated approaches for the simulation of the Na^+^-induced reduction in cell turgor pressure and related decrease in tissue expansion.

The discussed overestimation of leaf biomass explains the similar behavior showed by the model for the simulation of leaf area index (Fig. 2c) (CRM = −0.33), although the model correctly reproduced the relative reduction in leaf area for increasing Na^+^ concentrations (R^2^ = 0.89; EF = 0.65). This is considered as particularly important, since leaf area index is a key state variable involved with the amount of water daily transpired and thus with the potential entry of Na^+^ through the apoplastic pathway^17^ (equations 1–6).

Concerning Na^+^ uptake (Fig. 2d), simulated Na^+^ contents showed a good agreement with measured data, although the values of the performance metrics were slightly worse compared to those achieved for other outputs (RRMSE was around 50% and CRM was −0.20). However, the large portion of variance explained (R^2^ = 0.72) and the largely positive value of EF (0.66) allow considering also this variable as satisfactorily simulated. Indeed, Na^+^ content results from the simulation of both Na^+^ dynamics (entry, translocation, sequestration, etc.) and the related effects on crop growth, and also by the uncertainty of the crop model itself. The interaction between the crop model and the salt stress model is deeply involved with the way plant growth drives Na^+^ uptake through the simulation of actual transpiration (in turn driven by leaf area index) and Na^+^ sink capacity (driven by organ biomass). For these reasons, the general overestimation of plant Na^+^ content could be related to the overestimation in leaf area index and culm biomass (Fig. 2c and b). However, the model was not able to reproduce the high plant Na^+^ content measured at harvest for the cultivar Baldo at the highest Na level (150 mg plant^−1^). Another aspect able to partly explain the values for the agreement metrics obtained for the simulation of Na^+^ content is the Na^+^ accumulation for the control treatment (0 mM NaCl). In this case, the model simulates a null Na^+^ plant content, whereas small values were measured in real plant tissues.

### Identification of key traits in different scenarios

Simulations performed during the SA (5632 for each site) led to 14% and 12% average yield losses in California and Greece, respectively, in agreement with the expected yield reduction under salinity levels similar to those explored^47^. For the SA experiment in California, mean and standard deviation of simulated yields were 6523.26 kg ha^−1^ and 203.30 kg ha^−1^. Corresponding values for the Greek site were 7791.97 kg ha^−1^ and 280.41 kg ha^−1^.

**Figure 3:**
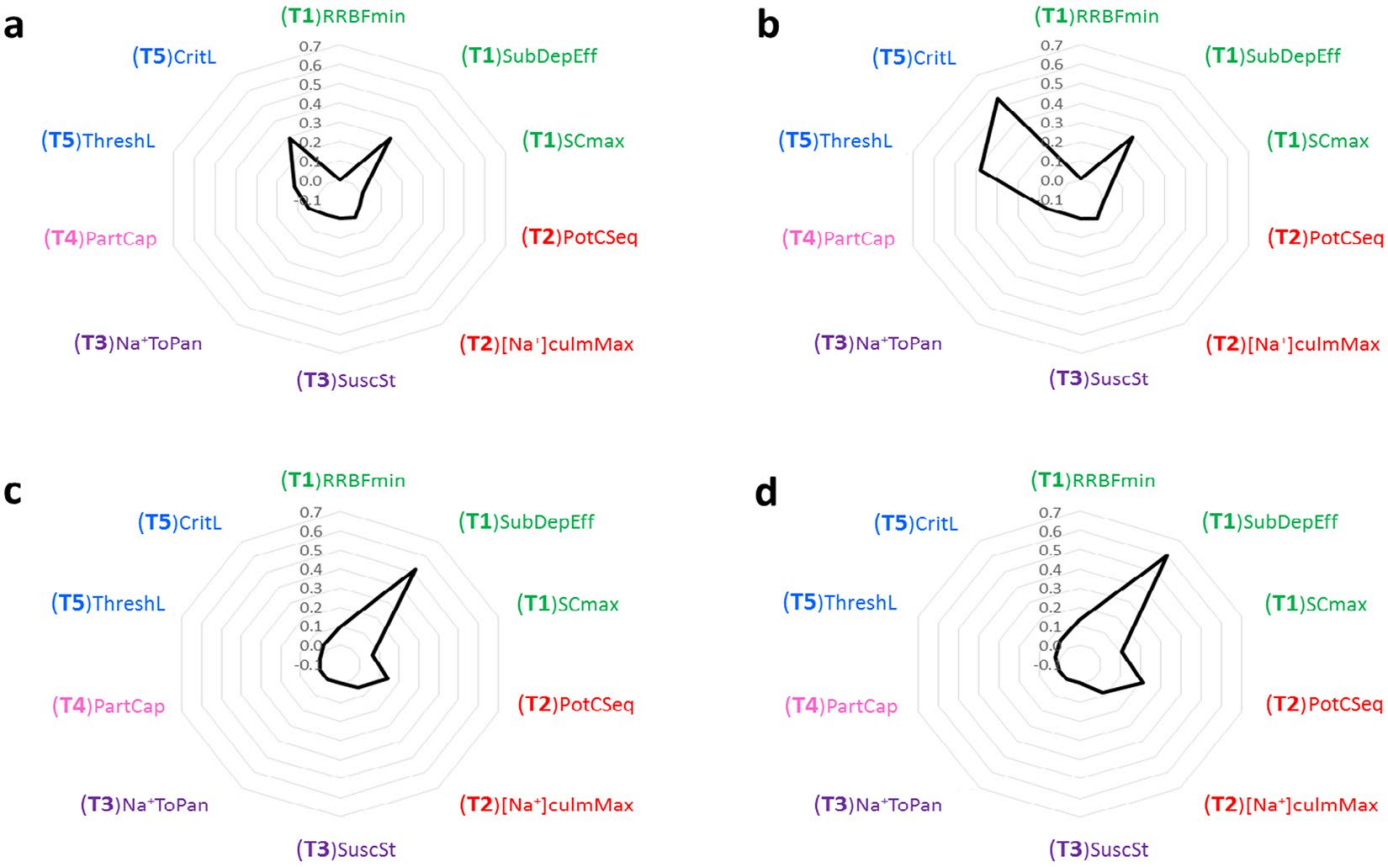
Sobol’ first- (**a**,**c**) and total-order (**b**,**d**) effects calculated for the sensitivity analysis performed on model parameters representing salt tolerance traits (Table 1). The Sobol’ sensitivity metrics for a parameter indicate the portion of output variance explained by changes in the value of that parameter alone (first-order effect) and of that parameter in interaction with all the others (total-order effect). The analysis was carried out for two scenarios, one in California (**a**,**b**) and the other in Greece (**c**,**d**). The output for which sensitivity metrics were calculated was the final yield.

**Figure 4:**
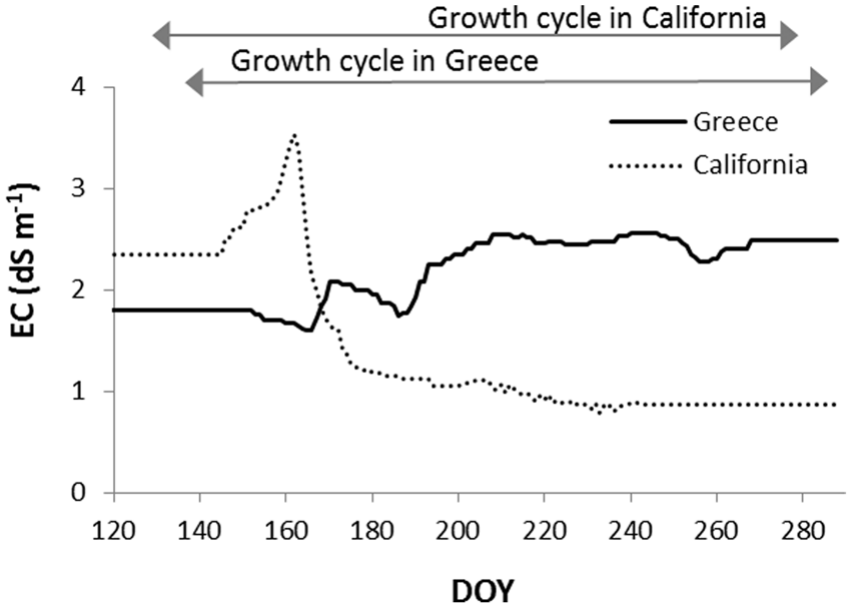
Seasonal electrical conductivity (dS m^−1^) of field water for the two scenarios used for the ideotyping study, derived from Scardaci *et al*.^51^ (California) and Lekakis *et al*.^54^ (Greece). Salinity dynamics follow different patterns: in the Californian site the highest level of salinity is reached in the first part of the crop cycle whereas in the Greek one salt concentration increases gradually until mid-season and remains constant afterwards.

Results of the SA (Fig. 3) showed how different scenarios could allow defining contrasting breeding strategies to increase the overall cultivar salt tolerance and, in turn, further demonstrated the potential of trait-based modelling for designing district-specific ideotypes^6^. Indeed, simulations revealed that – despite salt tolerance is an issue in both scenarios – different traits would guarantee the highest increase in yields in California and Greece. This is in agreement with Roy *et al*.^26^, who observed how different traits could be exploited to increase salt tolerance under different salinity levels. Tissue tolerance (T5, indicated in blue in Fig. 3) was the most important trait in California (Fig. 3b), where high-salinity peaks occur for short periods in the first part of the season (Fig. 4). Indeed, although the reduction of shoot Na^+^ uptake (T1, green in Fig. 3) also played a role (because of the relevance of a parameter involved with Na^+^ exclusion at root level via suberin deposition), the sharp increase in salinity in a moment when root barriers are still not developed makes the response at leaf level more important to increase the overall plant tolerance. A similar peak occurring later in the season (with a higher root suberin content) would have led to different results.

In case of prolonged stressful conditions like those characterizing the Greek scenario (Fig. 4), instead, the most important tolerance traits were involved with the plant capability to prevent Na^+^ from reaching leaf blades (Fig. 3d), i.e., reduction of Na^+^ uptake (suberin deposition) and − to a lesser extent – higher sequestration in structural tissues (T2, indicated in red in Fig. 3). The trait involved with tissue tolerance was not considered as relevant for this scenario, because Na^+^ accumulation in leaf tissues was so fast to rapidly overcome the capability of the plant to segregate toxic ions into the vacuole and to synthesize osmolytes. This is in agreement with Munns *et al*.^24^, who observed the same relationship between high salinity and the importance of excluding Na^+^ at root level for wheat.

The comparison of the Sobol’ first- and total-order effects highlighted strong interactions only for the Californian scenario (Fig. 3a and b) for the two parameters modulating the toxic effect of Na^+^ in leaf blades, i.e., (T5)ThreshL and (T5)CritL. This is partly due to the fact that these two parameters are involved in the same response function (equation 12), thus they interact with each other. However, the large differences in the values of the first- and total-order sensitivity metrics suggest that these two parameters interacted also with others, being tissue tolerance strictly depending on the whole chain of processes modulating the sodium uptake at root level and its translocation and accumulation in photosynthetic tissues.

Regardless of the scenario, parameters involved with tolerance to salt-induced spikelet sterility did not result important in affecting yields. The reason is that Na^+^ concentration in the young panicles was not high enough during the two sensitive stages (i.e., booting and flowering). In California, indeed, the accumulation of Na^+^ in the developing panicles before flowering was low because of the reduction of salinity due to fresh water inflow after the herbicide treatments, whereas in Greece Na^+^ concentration in field water started to increase too late to generate high contents in the reproductive organs before flowering.

For both California and Greece, the parameter values for the ideotypes were those that led to achieve the highest yield (i.e., those leading to the highest overall tolerance) during the SA experiments. Parameter values for the two ideotypes are shown in Table 2, together with the parameter values for the two cultivars used in the growth chamber experiments to evaluate the salinity model.

**Table 2:** Plant traits involved with tolerance to salinity and corresponding model parameters (acronym meanings and units are provided in Table 1) for the two rice cultivars for which growth chamber experiments were performed and for the ideotypes defined for the sites in California and Greece.

Traits	Parameters	Cultivars used in growth chamber experiments		Ideotypes	
		Vialone Nano	Baldo	California	Greece
Reduction of shoot Na^+^ uptake	(T1)RRBF_min_	5.0	5.0	4.7	4.7
	(T1)SubDepEff	0.62	0.62	0.66	0.64
	(T1)SCmax	30.0	30.0	31.3	30.2
Sequestration of Na^+^ in structural tissues	(T2)PotCSeq	0.028	0.030	0.030	0.029
	(T2)[Na^+^]culmMax	22	30	26	27
Tolerance to salt-induced sterility	(T3)SuscSt	0.0007	0.0020	0.0014	0.0014
	(T3)Na^+^ToPan	0.15	0.25	0.20	0.20
Compartmentation of Na^+^ into senescent leaves	(T4)PartCap	0.70	0.70	0.72	0.72
Tissue tolerance	(T5)ThreshL	1.0	2.0	1.4	1.4
	(T5)CritL	30.0	40.0	36.7	36.3

### Concluding remarks

Taking salt stress tolerance and rice as a case study, we showed how the design of ideotypes can benefit from the availability of models explicitly developed starting from traits breeders are working on. Indeed, the model developed demonstrated its suitability for analyzing in depth key G × E × M interactions. Salt-induced rice yield losses depend on the extent at which the genotype tolerance traits are effective in dealing with the salt stress dynamics deriving from management practices (e.g., water management modulating salinity level of rice field water) and weather conditions (e.g., evapotranspiration affecting potential sodium entry via by-pass flow). As our results show, these G × E × M interactions would lead the same genotype being tolerant in one environment and susceptible in another, as the ideotype defined for California, which would fail in coping with the prolonged salt-stress period of the Greek scenario.

However, salt tolerance is a complex trait relaying on a variety of physiological mechanisms that can be alternatively selected to improve the overall crop tolerance. The availability of new tools for genetic improvement (e.g., Marker Assisted Selection) allows breeding for specific traits instead of targeting the overall crop tolerance^26^, thus increasing the efficiency of the breeding process, since – as explained above – the effectiveness of the changes in the values of different traits varies according to the agro-environmental context.

For the first time, a modelling approach dedicated to ideotyping was developed by explicitly building algorithms around traits for which breeding activities are ongoing. We consider this strategy as the only one able to avoid inconsistencies between model parameters and plant traits, and thus between *in silico* ideotypes and the possibility of realizing them *in vivo*. Indeed, a high level of detail in the representation of physiological processes cannot be considered as a guarantee of direct relationships between model parameters and plant traits, given the same knowledge can be formalized in a variety of possible modelling structure^48^. Moreover, during model development, a pronounced process-based perspective was used to properly account for the key physiological processes and feedback mechanisms involved.

Results of model application to design district-specific ideotypes showed that, despite differences between scenarios were mainly limited to the seasonal dynamics of salt concentration in field water, putative traits to increase salt tolerance can rely on completely different physiological mechanisms. Results achieved thus encourage a paradigm shift towards the development of dedicated trait-based models and their use for supporting breeding programs at district level.

Limits of our study − and thus potential areas for model improvement − deal with the lack of approaches to simulate also the uptake and distribution of K^+^, since the toxicity of Na^+^ seems to be related also to the Na^+^: K^+^ ratio and not only to the concentration of Na^+^ in itself^18^. Also, improved (i.e., more explicit) approaches would be needed for the simulation of the effect of the osmotic component of salt stress on tissue expansion and stomatal reaction, in order to better account for osmotic adjustment.

## Methods

### The growth chamber experiments

Two rice (*Oryza sativa* L. spp. japonica) cultivars differing in their level of salt tolerance, i.e., Baldo and Vialone Nano, were grown in dedicated hydroponics growth chamber experiments. Caryopses were sterilized with 50% (v/v) Ca(ClO)_2_ for 30 min, thoroughly rinsed with deionized water and placed on wet filter paper at 26 °C in the dark for four days. Seven days old seedlings were then transferred to black plastic boxes containing the following nutrient solution^49^: 1.5 mM KNO_3_, 1 mM Ca(NO_3_)_2_, 500 μM MgSO_4_, 250 μM NH_4_H_2_PO_4_, 25 μM Fe-tartrate, 46 μM H_3_BO_3_, 9 μM MnCl_2_, 0.8 μM ZnSO_4_, 0.3 μM CuSO_4_, 0.1 μM (NH_4_)_6_Mo_7_O_24_, 30 μM Na_2_O_3_Si (pH 6.5). Floating polystyrene foam sheets were used to hold seedlings and to allow renewing solutions without touching the plants to avoid any potential damage to the roots (which would make Na^+^ entering directly from root lesions). Growing conditions were set to 14 h photoperiod; photosynthetically active radiation was supplied by fluorescent lamps (400 mmol m^−2^ s^−1^); day/night temperatures were 26 °C/18 °C; relative humidity ranged between 58 and 92%. Five NaCl treatments were applied from three weeks after sowing until maturity: 0 mM, 10 mM, 25 mM, 35 mM and 50 mM. In order to maintain NaCl concentrations nearly constant, solutions were renewed each three days.

At late heading (BBCH code 51) and maturity (BBCH code 92) three plants for each combination cultivar × treatment were harvested and divided into stems, panicles and leaves. The latter were further separated into apical, medium and senescent leaves (referring respectively to the two youngest leaves, other green leaves and dead ones) to detect potential variation in Na^+^ accumulation among leaves of different ages. Plant height, number of tillers and dry biomass of each organ were measured. Dry biomass samples were ground to a fine powder and digested by concentrated HNO_3_ (10 mM) in a microwave digester (ETHOS D, milestone, Italy) at 100 °C^50^. The mineralized material was dissolved in 5 mL 0.1 M HNO_3_ and Na^+^ content was measured by inductively coupled plasma mass spectrometry (Bruker Aurora M90 ICP-MS, ICP Mass Spectrometer). Na^+^ content and dry biomass of different plant organs were then used to calculate corresponding Na^+^ concentrations. Immediately before the first sampling event (late heading), the impact of salt stress on net photosynthetic rate, stomatal conductance and transpiration rate was measured on the youngest fully expanded leaf using a CIRAS-3 Portable Photosynthesis System (PP Systems, Amsbury, MA, USA). Apical and medium leaves were then scanned to determine plant leaf area and SLA, the latter calculated as ratio between leaf area and leaf dry biomass. At harvest, spikelet sterility was determined.

### The ideotyping study

#### Case studies and simulation scenarios

An ideotyping study was carried out to using the salt stress model to identify the traits breeders should focus on in two production areas differing for the salinity seasonal dynamics (Fig. 4). Colusa is one of the six counties at the north of Sacramento where the production of rice in California ‒ the second largest rice-producing state in the US ‒ is concentrated. While most of the irrigation water has a low salt content, water holding periods for herbicide distribution and high temperatures promote evapo-concentration of salt in rice fields^51, 52^. This leads salinity in field water during the first part of the crop cycle to increase up to 3.5 dS m^−1^, and to decrease rapidly once the flow of fresh water is restored. Although the mean seasonal salinity is not high, the rice susceptibility during early phenological phases can lead yield losses to exceed 10%^47^. The second scenario targets the southeastern region of Axios River plain, near Thessaloniki, one of the key regions for rice production in Greece^53^. Ninety percent of the soils in the area are saline, causing increases in the salts content of irrigation water during infiltration. Evapo-concentration of salts due to high temperatures also contributes to increase salinity, which progressively reaches values of 2.5 dS m^−1^from mid-season to harvest. Dynamics of salinity in field water and information on management practices used for the two case studies were derived from Scardaci *et al*.^51^ and Linquist *et al*.^52^ (California), and from Lekakis *et al*.^54^ (Greece).

Simulations were performed for the cultivar Thaibonnet (also known as L202), developed by the California Co-operate Rice Research Foundation and currently representing an important variety in Greece^53^.

#### Sensitivity analysis

The ideotyping study was performed using global sensitivity analysis (SA) techniques^4, 5^. In particular, the variance-based method of Sobol’^55^– considered as a references for SA^56^ – was used, targeting yield as reference output. The analysis focused on first- and total-order effects, accounting, respectively, for the effects of variations in each parameter on simulated yield, and for the effects of variations in parameter including possible interactions with others. The sample size for the combinations of parameters was 5632, i.e., the lowest value of *M* | *M* > (*γ* · *n*), with *M* = 2^*q*+3^(2*n* + 2), *q* = {1, 2, 3, …, *Q*}, *γ* is the suggested number of model runs for each parameter, and *n* is the number of parameters in the sensitivity analysis. In this study, *γ* was set to 500^57^.

Parameterization of the crop model WOFOST-GT2 for Tropical Japonica rice varieties was derived from Stella *et al*.^44^. Concerning the parameters of the salt stress model, the values derived from the growth chamber experiments were used as means (Table 1), with the exception of a correction factor applied to the maximum suberin content to account for differences in root development between hydroponic and soil conditions^58^. Distributions for the SA were assumed as normal, and standard deviations were set to 5% of the mean values for the parameters^59^.

In order to avoid the risk of including in SA results the effect of a specific meteorological season, simulations for both sites were performed on 25-year series of weather data (1990–2015) retrieved from the European Centre for Medium-Range Weather Forecasts (ECMWF; ERA-Interim database; www.ecmwf.int).
